# Genetic architecture and temporal patterns of biomass accumulation in spring barley revealed by image analysis

**DOI:** 10.1186/s12870-017-1085-4

**Published:** 2017-08-10

**Authors:** Kerstin Neumann, Yusheng Zhao, Jianting Chu, Jens Keilwagen, Jochen C. Reif, Benjamin Kilian, Andreas Graner

**Affiliations:** 1 Leibniz Institute of Plant Genetics and Crop Plant Research (IPK), Seeland OT Gatersleben, Germany; 2 Julius Kühn-Institute (JKI), Federal Research Centre for Cultivated Plants, Quedlinburg, Germany; 3 Global Crop Diversity Trust (GCDT), Bonn, Germany

**Keywords:** Barley, Development, Genetic architecture, Genome-wide association mapping, Growth, High-throughput phenotyping, Non-invasive, Photoperiod, Vegetative biomass

## Abstract

**Background:**

Genetic mapping of phenotypic traits generally focuses on a single time point, but biomass accumulates continuously during plant development. Resolution of the temporal dynamics that affect biomass recently became feasible using non-destructive imaging.

**Results:**

With the aim to identify key genetic factors for vegetative biomass formation from the seedling stage to flowering, we explored growth over time in a diverse collection of two-rowed spring barley accessions. High heritabilities facilitated the temporal analysis of trait relationships and identification of quantitative trait loci (QTL). Biomass QTL tended to persist only a short period during early growth. More persistent QTL were detected around the booting stage. We identified seven major biomass QTL, which together explain 55% of the genetic variance at the seedling stage, and 43% at the booting stage. Three biomass QTL co-located with genes or QTL involved in phenology. The most important locus for biomass was independent from phenology and is located on chromosome 7HL at 141 cM. This locus explained ~20% of the genetic variance, was significant over a long period of time and co-located with *HvDIM*, a gene involved in brassinosteroid synthesis.

**Conclusions:**

Biomass is a dynamic trait and is therefore orchestrated by different QTL during early and late growth stages. Marker-assisted selection for high biomass at booting stage is most effective by also including favorable alleles from seedling biomass QTL. Selection for dynamic QTL may enhance genetic gain for complex traits such as biomass or, in the future, even grain yield.

**Electronic supplementary material:**

The online version of this article (doi:10.1186/s12870-017-1085-4) contains supplementary material, which is available to authorized users.

## Background

Increases in both yield and yield stability are key objectives in plant breeding to support an ever expanding population [1]. Grain yield and yield stability are complex traits, and their genetic improvement has been impaired by notoriously low field plot-based heritability. Consequently, during the past two decades breeding efforts to increase barley yields have made minimal progress while grain yields have stagnated in Europe and other regions [2]. Losses of 15 to 22% in yield have been projected for this crop due to the effects of climate change [3].

Grain yield potential can be improved by enhancing either sink or source strength. Enhancing sink strength results in a further increase in harvest index, partitioning assimilates towards the grain; enhancing source strength may require an increase in vegetative biomass [4]. Most of the historical increases in barley yield reflect changes in harvest index (weight of grain divided by weight of above-ground biomass), an effect of enhanced sink strength, while overall biomass has remained unchanged [5–7]. As one consequence, the harvest index has come close to a proposed upper limit of 0.6 [8]. Other reports suggest a positive relationship between biomass and grain yield [9–11] and thus indicate that increasing biomass may be a promising approach for improving grain yields in barley as it was recently recommended for wheat [12].

Automated high-throughput phenotyping (HTP) has evolved quickly and offers a non-destructive, image-based method for the analysis of complex traits [13]. Previously, cumbersome and destructive measurements of above-ground biomass, targeting a defined developmental stage, enabled only end-point analyses [14]. By contrast, phenotyping conducted throughout the plant’s life cycle allows crop growth to be tracked over time. The feasibility of image-based biomass assessment has been reported for a wide variety of crops and plants with different architectures, including arabidopsis*,* maize, soybean, wheat, and barley [15–17]. Daily, non-destructive estimation of biomass over the vegetative growth period revealed logistic-like biomass accumulation under greenhouse conditions [18] with most of the vegetative biomass forming prior to flowering. The logistic model can be used to identify the time point of maximum growth, which has been supposed to be linked to developmental speed and potentially with flowering time [19, 20]. Data from the logistic growth model for barley have also been shown to provide high heritabilities for biomass and secondary traits [20], enabling the analysis of the genetic architecture of biomass development.

Genome-wide association studies (GWAS) allow the analysis of a wide range of genetic and phenotypic diversity in a single population and therefore GWAS have been widely employed for quantitative trait analysis [21–23]. Combining GWAS with non-destructive trait assessment identified time-specific QTL for biomass in triticale [24] and maize [25].

The goal of our study was to elucidate the key genetic factors controlling biomass accumulation and to resolve their temporal dynamics using GWAS. To this end, we performed image-based phenotyping of a diverse set of two-rowed spring barley lines throughout their vegetative growth.

## Methods

### Germplasm and experimental set-up

A set of 97 lines from the spring barley collection, described by [26, 27], was used in the biomass assays. To minimize population stratification and the effects of phenology, only two-rowed accessions were chosen, mainly of European origin, and very early and late genotypes were excluded. The range of average flowering time was 9 days in the selected subset. The cultivars in the collection were released between 1924 and 1990 with the majority originating from 1960 to 1980. Our panel also included three additional cultivars that were not part of the above-mentioned collection (Additional file 1: Table S1). The 100 genotypes were grown in a greenhouse equipped with a LemnaTec-Scanalyzer 3D system (LemnaTec GmbH, Aachen, Germany), holding a total of 520 pots on a conveyor belt system. Three consecutive experiments were performed between May and November 2012, (Additional file 1: Table S2) each with five replicates per genotype. Each experiment lasted 58 days - up to the beginning of the reproductive stage according to the established experimental design in [20]. No fertilizer was applied, but whenever necessary, plants were sprayed against fungal diseases and aphids. Pots were watered daily to a target weight corresponding to 90% field capacity. Greenhouse temperature was set to 18 °C during the day and 16 °C during the night. Pot size, soil, and light conditions were as described in [20]; with the exception that illumination was prolonged from 13 to 15 h per day. All seeds used in this study, including those used for measurement of thousand-kernel weight (TKW), originated from a field trial at the Leibniz Institute of Plant Genetics and Crop Plant Research (IPK) in 2011. Two seeds per replicate of each genotype were sown directly into the pots on the system, and thinned to one seedling per pot after 7 (experiments 1 and 2) or 9 (experiment 3) days after sowing (DAS). Plants were fully randomized each night to overcome any potential inhomogeneity within the greenhouse in terms of light and temperature distribution.

Daily imaging in the visible light range started at 10 DAS with one top view image and three side view images at 0°, 45°, and 90° collected for each plant. The resolution of the digital camera (Basler AG, Germany) was 1628 × 1236 pixels, with a pixel size of 4.4 × 4.4 μm. Technical issues resulted in a loss of images or incomplete sets of images for a total of 7 days across all three experiments (Additional file 1). Images were exported and analysed using the Integrated Analysis Platform (IAP) [28]. Using side and top view areas, a volume (unit: voxel) termed ‘digital biomass’ was calculated and used as a proxy for biomass [20].

The images taken at 58 DAS were visually inspected and the growth stage was scored using the BBCH-scale [29, 30]. For plants that reached BBCH 49 (tip of awn visible) prior to 58 DAS, the exact date of tipping time (time of awn emergence at flag leaf) was determined by visual inspection of the earlier images. In the early morning of 59 DAS, above-ground biomass was measured as fresh weight. The number of tillers was manually counted at 27, 45, and 58 DAS.

### Phenotypic analysis

As plants were fully randomized each night, we considered the experimental design as a completely randomized design for statistical analysis. All statistical analyses were performed in R [31]. Digital biomass was analyzed from 10 to 58 DAS and tiller numbers for 27, 45, and 58 DAS counts were analyzed. An outlier test following [32] was performed each day within all three experiments. Altogether, less than 1% of the data points were considered outliers.

We performed a two-step analysis of the phenotypic data. In the first step, best linear unbiased estimates (BLUEs) were calculated for each day, within each experiment, with the model *Y = G + e*, where *Y* is the phenotypic value of a trait for each plant, *G* represents the fixed effect of the genotype and *e* the residual error (errors were assumed to be normally, independently, and identically distributed). In the second step, BLUEs were estimated across environments by fitting the model *Y = G + E + e*, assuming that all effects except genotype were random. Here, *Y* refers to the BLUE estimated in the first step for each trait, *G* the effect of genotype, *E* the effect of experiment, and *e* the residual error. Moreover, we performed a one-step model to estimate the phenotypic variance components by fitting the model *Y = G + E + GxE+ e*, assuming that all effects were random effects.

We used the BLUEs of single experiments for digital biomass from 10 to 58 DAS and for each plant fitted a logistic growth model as $f(t)=\frac{a}{1+b{e}^{- ct}}$, where *f*(*t*) is the digital biomass at time point t. The inflection point was determined as ${t}_0=\frac{\log (b)}{c}$. The parameters were estimated using Matlab software (MathWorks, Inc., MA, United States) [33, 34].

Broad sense heritability was calculated as

$$ {H}^2=\frac{V_G}{V_G+\frac{V_{GE}}{O}+\frac{V_e}{OR}}, $$

where *V*_*G*,_ *V*_*GE*_, and *V*_*e*_ are the variance components of the genotype, genotype x experiment and the residual, respectively. *O* is the number of experiments for the respective DAS, and *R* the number of biological replicates. Further, we assumed fixed genotypic effects and estimated overall BLUEs.

### Genotyping

SNP genotyping using the 9 K iSelect array (Illumina, CA, United States) was performed as described in [35]. From a total of 7864 SNP assays performed on the barley collection, a set of 4866 SNPs were polymorphic, with a minor allele frequency (MAF) >0.05 and less than 5% missing data. A number of 3041 SNPs were mapped using the POPSEQ approach [36] and additional 1081 SNPs were mapped using information from the Morex x Barke (MxB) recombinant inbred line (RIL) map [35]. The remaining 744 SNPs lacked genetic positions. Based on marker analysis, one accession (BCC1367) was removed from genetic analysis, because its authenticity could not be clearly determined, leaving 99 genotypes for association analysis.

Linkage disequilibrium (LD) was estimated as squared correlation coefficient (r^2^) for all mapped markers on individual chromosomes. LD decay by genetic distance was plotted and a LOESS curve fitted to the data points. The 95th percentile of r^2^ of all unlinked intrachromosomal marker pairs (>50 cM distance) was estimated according to [37] and used to obtain a population-specific threshold for genome-wide LD due to linkage. The intercept of this r^2^-threshold and the LOESS curve determines the extent of LD from linkage.

### Genome-wide association study

GWAS was performed using BLUEs from single experiments. The following mixed-linear model was applied to the daily data of digital biomass, the calculated inflection point, tiller number, and fresh weight at experiment end:

*Y* = μ + *E* + *S* + *G* + *e*,

where μ is the overall mean and *E* is the effect of experiments, *S* is the effect of SNP and *G* is the random effect of genotype, while *e* is residual error. This model has covariance structure of 2 K*σ*_*G*_^2^, where *K* refers to the kinship matrix [38] and *σ*_*G*_^2^ is the genetic variance. A false discovery rate (FDR) with a significance level of 0.1 was applied. The proportion of genetic variance of the detected QTL was estimated as the adjusted r^2^ values standardized with the heritability. Association analyses were performed using the software ASReml-R 3.0 [39].

### Computer simulations

A simulation study was conducted to verify that QTL with large effects could be detected in our mapping population and is described in detail in [38]. We randomly selected two markers and set them as artificial QTL with different effect levels. The markers explained 15% and 10% of the genetic variation, respectively. We applied the described GWAS in the simulated data and evaluated the detection rate of the two QTL. The simulation was repeated 100 times.

## Results

### High heritabilities for all examined traits

**Fig. 1:**
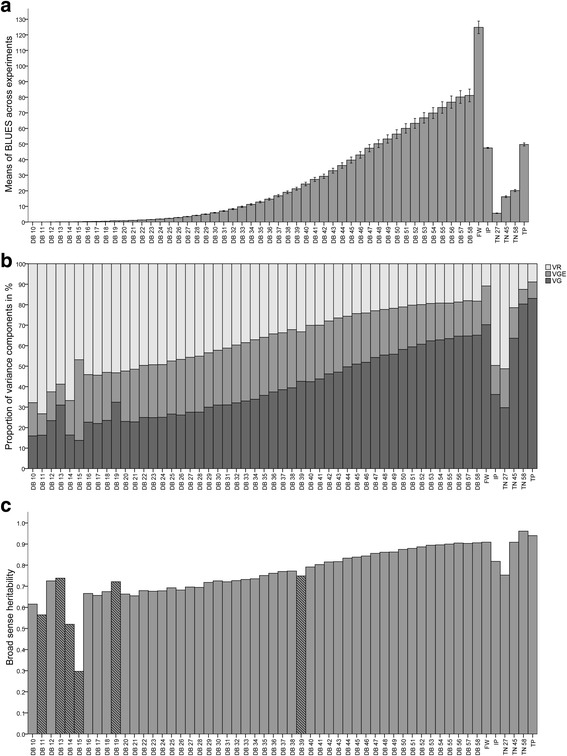
Overview of BLUEs, variance components, and broad sense heritability of estimated traits for 100 barley lines. **a** Bar diagram of the overall BLUEs for all analyzed traits: digital biomass (DB) from 10 to 58 days after sowing (DAS) in 10^6^ Voxel, fresh weight (FW) in g estimated at DAS 59, inflection point (IP) in DAS, tiller number (TN) at DAS 27, 45 and 58 and tipping time (TP). Error bars represent the 95% confidence interval. **b** Proportion of variance components in % for each time point of DB (DB10 to DB58), FW, IP, TN (TN 27, 45 and 58) and TP, where VG refers to genotypic variance, VGE to genotype x environment interaction and VR the variance of the rest (unexplained variance). **c** Bar diagram presenting broad sense heritability values for DB (DB10 to DB58), FW, IP, TN (TN 27, 45 and 58) and TP. Hatched bars represent days with missing data

The barley diversity panel was evaluated for tiller number, tipping time, biomass over time, and inflection point, i.e. the time point of maximum growth (Fig. 1a, Additional file 1: Figures S1, S2, S3). Except for inflection point, all traits showed broad phenotypic variation resulting in high coefficients of variation (Additional file 1: Table S3). Variance component analyses of biomass over time (Fig. 1b) revealed that heritability increased from 0.62 during the seedling stage to a maximum of 0.91 at the late booting stage (Fig. 1c). The same trend was observed for tiller number. Moreover, heritability was high for inflection point (0.82) and tipping time (0.96). In summary, the intensive phenotyping of the barley diversity panel resulted in high-quality phenotypic data forming a solid basis for genetic analysis.

### Dynamic phenotyping revealed substantial genotype-by-time interactions

**Fig. 2:**
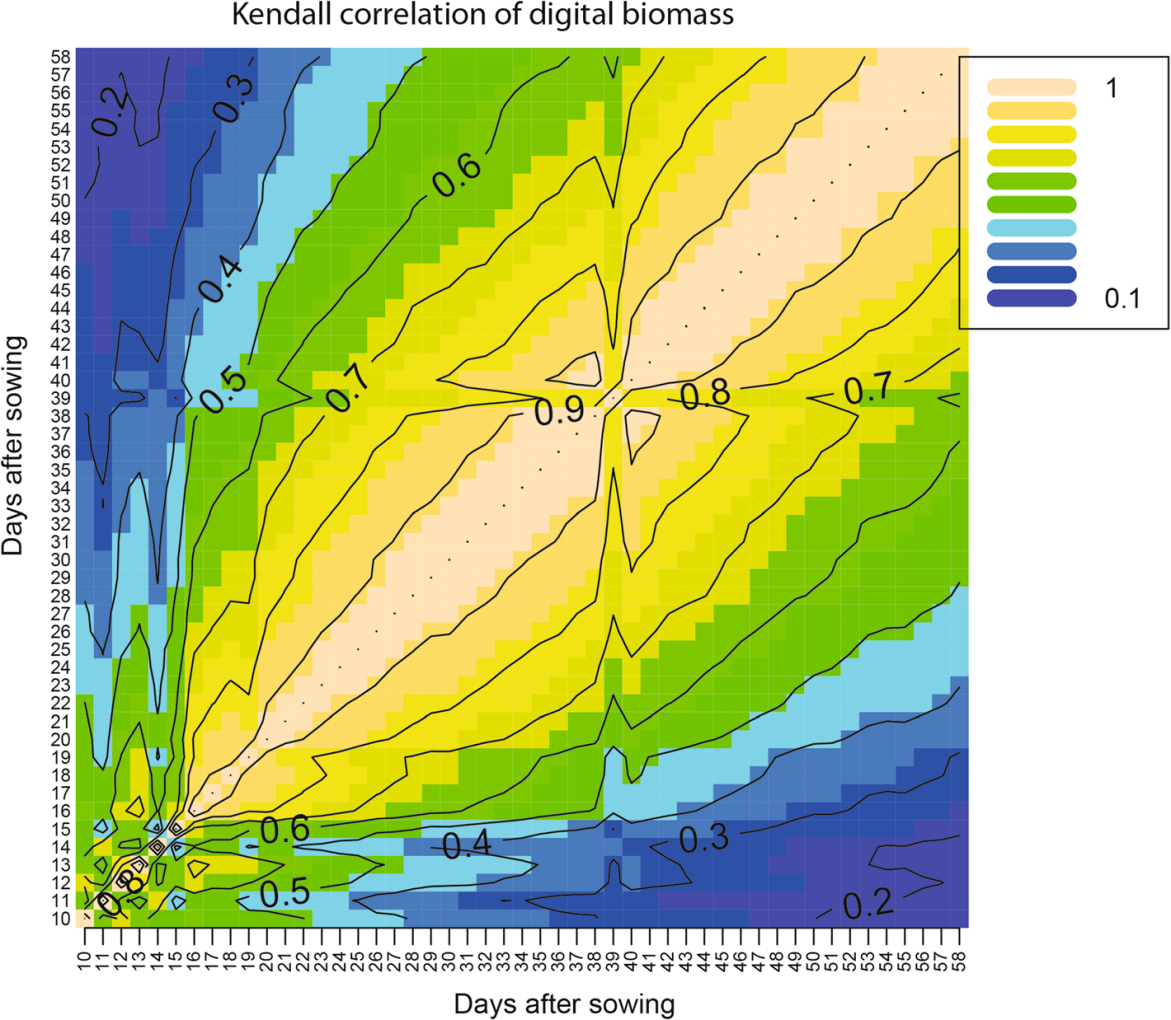
Heatmap for Kendall rank correlation coefficients between BLUEs of digital biomass from days after sowing (DAS) 10 to 58. Colors range from *purple* (for correlation ~ 0.1) to *light-yellow* (for correlation ~0.9). Numbers in the contour line indicate the level of correlation

We observed that image-based digital biomass measurements are a precise proxy for manually measured fresh biomass (Additional file 1 Figure S4), and facilitate plant growth assessments. Evaluation of trends in image-based digital biomass revealed that genotypes were characterized by rapidly changing patterns of biomass accumulation during early growth stages (Fig. 2). This is reflected by the low Kendall rank correlations of digital biomass during the seedling stages (Fig. 2, lower left quadrant). As plants matured, higher correlations among adjacent time points were observed (Fig. 2, upper right quadrant). More distant time measurements, as between the seedling and booting stages, were not correlated (*r* = 0.1; *P* > 0.05) pointing to strong genotype-by-time interactions.

### Associations between biomass and other agronomic traits

Heterotrophic and early autotrophic growth is often dependent on the initial seed weight. According to this expectation, we observed a moderate correlation between digital biomass at seedling stage and TKW (*r* = 0.41; *P* < 0.001; Additional file 1: Figure S5).

Phenology can also substantially affect plant growth. Tipping time is one important factor of phenology. Thus, we inspected the association between tipping time and inflection point to describe the dynamics of biomass development. We observed a moderate correlation (*r* = 0.45; *P* < 0.001; Additional file 1: Figure S6) between these traits. The correlation between tipping time and final biomass was more pronounced at 0.58 (*P* < 0.001).

Biomass is expected to increase in concert with tiller number. In accordance with this expectation, we observed that digital biomass significantly correlated with the number of tillers. For the three time points at which tiller number was assessed, correlation coefficients exceeded 0.5 (Additional file 1: Figure S5, Table S4).

### Linkage disequilibrium and population structure

After filtering for minor allele frequency and missing data, a total of 4866 SNPs were used for further analyses. Out of these, a genetic map position had been assigned to 4122 SNPs, providing good coverage across the 7 barley chromosomes (Additional file 1: Figure S7). The average linkage disequilibrium decay in our panel of spring barley lines amounted to 8 cM, but significant variation was observed among individual chromosomes (Additional file 1: Figures S8, S9).

Population stratification can lead to an inflated rate of false-positives in GWAS. The present panel was deliberately selected to avoid the major causes of population structure such as growth habit, row type or origin. Applying a principal coordinate analysis (based on all 4866 SNPs) this is substantiated by the small amount of molecular variance explained by the first two PCs (16%, Additional file 1: Figure S10). After examining population structure at higher resolution, family structures could be detected. Here, groups of accessions with common progenitors, such as eight cultivars of which six descend at different pedigree levels from the old German cultivar Isaria (BCC1391), cluster in one clade (Additional file 1: Figure S11). Another example is a cluster of four genotypes originating from Syria. Therefore, a kinship matrix was used in the GWAS to correct for population stratification.

### Identification of major biomass QTL using GWAS

**Fig. 3:**
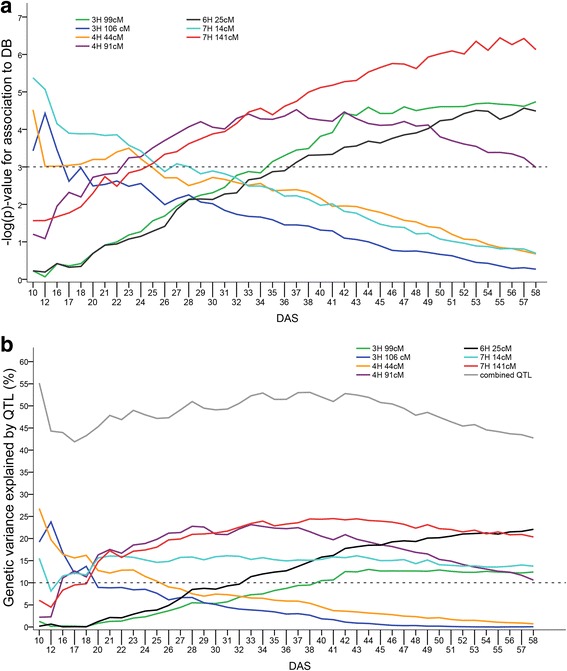
Time course of QTL-dynamics for digital biomass (DB) in days after sowing (DAS). The figure represents seven SNPs that exceeded the FDR threshold of 0.1 for at least 1 day. Note that missing DAS data points were excluded in this graph. **a** Significance value –log(p) over time - each colored line represents one QTL. **b** Proportion of genetic variance explained by QTL separately (*lower, colored lines*) or in combination (*upper grey line*)

A simulation study was conducted to validate the statistical power to detect QTL with large effects in our mapping population. QTL that explained at least 15% of the genotypic variation were detected in 58% of the simulation runs. Hence, the population size is considered large enough to detect major QTL. A total of seven SNPs, representing seven different loci, surpassed the FDR threshold of 0.1 for biomass in at least one time point (Fig. 3a, Additional file 1: Figure S12, Additional file 2). We observed three distinct trends for significant biomass-marker associations over time: monotonic increases, monotonic decreases, and an increase followed by a decrease. More specifically, these include: 1) three SNPs on 3H 106 cM, 4H 44 cM, and 7H 14 cM decreased monotonically over time; 2) three SNPs mapping to 3H 99 cM, 6H 25 cM, and 7H 141 cM showed a monotonic increase in –log(p)-values over time; 3) –log(p)-values of one SNP located at 4H 91 cM increased until DAS 37 and decreased thereafter. These seven biomass QTL collectively explained between 42% of the genetic variation at 17 DAS and 55% of the genetic variation at 10 DAS (Fig. 3b). Individual QTL explained between 13% and 27% of the genetic variation.

Two SNPs located on chromosome 1H at 71 cM and on chromosome 7H at 34 cM showed significant associations with the inflection points (Additional file 1: Figure S13, Additional file 2). The two SNPs explained 9% (7H) and 8% (1H) of genetic variation respectively, and 16% of the genetic variation collectively.

### Marker-trait associations for tipping time and tiller number

One SNP (SCRI_RS_140819) on chromosome 2H at 27.7 cM near *PPD-H1* (19.9 cM) showed a significant association with tipping time (Additional file 1: Figure S14, Additional file 2), explaining 23% of the genetic variation. *PPD-H1* is an important regulator of photoperiod response determining flowering in barley and the genomic region of *PPD-H1* was harboring the main heading time QTL in the full barley panel [27]. Although the functional SNP of *PPD-H1* (*BK_15*) is part of the used marker set, it did not pass the FDR (−log(p) value was 2.4). The functional SNP showed significant linkage disequilibrium of r^2^ = 0.34 to the SNP at 27.7 cM. A higher minor allele frequency of the more distant SNP (MAF = 0.152) compared to BK_15 (MAF = 0.101) may be the reason for not detecting the functional SNP as an association. However, the proportion of genetic variance explained by *BK_15* is high (24%) despite its lacking significance and therefore the QTL is congruent with the earlier findings [27].

No SNP was significantly associated with tiller number at 27 DAS (Additional file 1: Figure S15, Additional file 2). In contrast, 31 significant marker-trait associations were detected for tiller number at 45 DAS. The SNPs comprised seven different loci and collectively explained 36% of the total genetic variation. Three SNPs located on chromosome 6H at 30 cM explained the highest amount of genetic variation (11%). Tiller number assessed at 58 DAS was significantly associated with 42 SNPs mapping to ten different loci. The 42 SNPs explained collectively 54% of the genetic variation. The same three SNPs identified in the 45 DAS tiller data set on chromosome 6H at 30 cM, explained most of the genetic variation (20%).

## Discussion

In this study we investigated above-ground biomass formation during vegetative phases of plant development in a diverse panel of two-rowed spring barley accessions using GWAS of image- and model-based trait components. This barley collection has been investigated in several other genetic analyses (Table 1) and allows direct comparison of our results to those obtained by [40–42, 27], who investigated the full panel of 224 spring barley accessions at flowering, seedling, and at maturity stages, respectively. From this panel, 96 two-rowed barley genotypes were analyzed for GWAS in this study, allowing comparisons between our study and these results. Alqudah et al. [40] investigated differential developmental phase duration QTL, which we refer to as stage duration QTL. Alqudah et al. [41] investigated plant height and tiller number in the two-and six rowed barley panel and we compared our results only with their QTL in the two-rowed panel.

**Table 1:** Overview of genetic loci identified by GWAS

Chr	Position (cM)	Trait	Published candidate loci in target region	QTL in other studies using iSelect or BOPA arrays
1H	70.5	IP		
2H	27.7	TP	*PPD-H1*	Alqudah et al. (2014): stage duration; Pasam et al. (2012 and (Maurer et al. 2015, 2016): heading; height, TKW, starch, protein; Ingvordsen et al. (2015): grain yield (23.0 cM), Sannemann et al. (2015) heading
2H	57.4–58.1	TN58	*Eps2/eam6/HvCEN* (58 cM)	Alqudah et al. (2014): stage duration; Alqudah et al. (2016): TN; Long et al. (2013) and Tondelli et al. (2013): height; Maurer et al. (2015): flowering; Maurer et al. (2016): shooting, shoot elongation phase, heading, ripening, maturity, height; Pasam et al. (2012): heading, height
2H	74.4	TN 58		Alqudah et al. (2014): stage duration
2H	124.9	TN 45 + 58	*HvAP2* (126.7 cM)	Maurer et al. (2016): shooting, hedensading, ripening (127–130 cM); Pasam et al. (2012): height
2H	135.8	TN58		Alqudah et al. (2014): stage duration; Maurer et al. (2016): maturity, height (139 cM); Pasam et al. (2012): height; Tondelli et al. (2013): height
3H	98.7	DB 42–58, FW	*HvCMF1* (98.2 cM)	Ingvordsen et al. (2015): grain yield (100.3 cM); Maurer et al. (2016): shooting, shoot elongation phase, heading, ripening, maturity, height, TKW; Long et al. (2013): root weight; Wehner et al. (2015): biomass yield
3H	105.9	DB 12		Alqudah et al. (2016): TN; Maurer et al. (2015): flowering (107.8–109.2 cM); Maurer et al. (2016): shooting, shoot elongation phase, heading, ripening, maturity, height, TKW; Sannemann et al. (2015): heading; Wehner et al. (2015): biomass yield
4H	43.6	DB 10		Ingvordsen et al. (2015): straw biomass
4H	91	DB 33–47		
5H	30.6	TN58		
5H	42.0–45.7	TN 45 + 58	*HvCO3* (43.7 cM) *HvTFL1* (44.1 cM), *HvCMF13* (46.4 cM)	Alqudah et al. (2014): stage duration; Alqudah et al. (2016): height; Maurer et al. (2016): shoot elongation phase; Pasam et al. (2012): height; Wehner et al. (2015): biomass yield
6H	24.5	DB 50–58, FW, TN 45		Alqudah et al. (2016): TN
6H	30.1–30.2	TN 45 + 58		
6H	55	TN45	*HvCO5/HvCry1a/* *HvCry2/HvPRR1/HvTOC1* (55 cM)	Alqudah et al. (2016): TN, height; Maurer et al. (2016): shooting, heading, ripening, height; Pasam et al. (2012): height, TKW; Tondelli et al. (2013): lodging
7H	14.0	DB 10–12		Alqudah et al. (2014): stage duration, Alqudah et al. (2016): TN; Maurer et al. (2016): shooting; Long et al. (2013): TN
7H	34.0	IP	*Vrn-H3/HvFT1*	Alqudah et al. (2014): stage duration; Maurer et al. (2015), Pasam et al. (2012), Rollins et al. (2013) and Sannemann et al. (2015): heading/flowering; Maurer et al. (2016): shooting, shoot elongation phase, heading, ripening, maturity, height, TKW
7H	67.8–68.1	TN 45 + 58	*HvCO1* (67.9 cM), *HvCO5* (70.5 cM)	Alqudah et al. (2014): stage duration; Alqudah et al. (2016): TN, height; Maurer et al. (2016): shooting, shoot elongation phase, heading, maturity, height, TKW; Pasam et al. (2012): heading, height; Sannemann et al. (2015): heading; Wehner et al. (2015): biomass yield (70.2 cM)
7H	120.4	TN 45 + 58	*HvCO6* (120.8 cM)	Alqudah et al. (2014): stage duration; Alqudah et al. (2016): height; Ingvordsen et al. (2015): yield variance; Maurer et al. (2016): shooting, heading, ripening, maturity; TKW Tondelli et al. (2013): height
7H	134.2	TN58		Wehner et al. (2015): biomass yield (133.9 cM)
7H	140.9	DB 33–58, FW	*HvDIM*	George et al. (2014): shoot dry weight

SNPs are described by their genetic position and associated traits. Candidate genes for flowering traits are based on positions from Alqudah et al. (2014) and comparison to growth and heading/flowering related traits from other mapping studies in barley that used the same barley collection and/or SNPs from iSelect or barley oligo pool array (BOPA)

*IP* inflection point, *TP* tipping time, *DB* digital biomass, *FW* fresh weight, *TN* tiller number (at a specific number of DAS)

Moreover, the 9K iSelect array has also been used in applied genomic research [35], allowing additional comparisons of QTL in other populations. George et al. [43] investigated a European spring barley collection in the juvenile stage. Ingvordsen et al. [44] investigated a Nordic spring barley collection at maturity stage. Maurer et al. [45, 46] investigated flowering time and plant development in a barley NAM-population; Rollins et al. [11] investigated a Syrian spring barley RIL population at maturity stage; Sannemann et al. [47] investigated a German two-rowed barley MAGIC population for flowering time; Tondelli et al. [22] investigated a set of 116 European two-rowed barley cultivars until maturity stage; Wehner et al. [48] investigated a German and Spanish winter barley collection in the juvenile stage.

In total, 17 out of 21 loci for biomass and related traits in our study were co-locating with QTL from the previous mentioned studies (Table 1).

### Plant growth was assessed with high precision enabling dynamic association mapping

The observed heritability estimates of image-based biomass (Fig. 1c) were high, reaching 0.9 in later stages, and similar to those identified previously in barley [15, 20]. Interestingly, the observed heritability for the inflection point (H^2^ = 0.82) was substantially higher than that seen in a previous wheat study that reported a heritability estimate of 0.07 [19]. The strict control of our environmental conditions, across the entire growing period, may have been key to achieving the high heritability results for biomass traits. Recently a high heritability of 0.72 was also observed in a large maize panel in strictly controlled conditions [25]. The heritabilities facilitated the identification of key genetic factors underlying biomass development. Moreover, these heritabilities will enable a reduction in the number of genotype replicates required and facilitate phenotyping larger populations in future studies. This will be advantageous for detecting QTL with smaller effects since both the sensitivity and the selectivity of GWAS analysis increase with population size [49]. The obtained heritabilities facilitate future screening of larger collections of 200 genotypes on this platform suitable for resolving smaller-effect QTL by further decreasing the replicate number down to two replicates: according to the variance component analysis, based on two replicates and three experiments, a heritability of ~0.5 can still be achieved for seedling biomass, while biomass around reproductive stage is projected to be 0.9.

### The genetic architecture of biomass is partially driven by phenology and morphology

The positive correlation (*r* = 0.58) between final biomass and tipping time (Additional file 1: Figure S6) revealed that a prolonged vegetative growing phase promotes higher biomass accumulation. The relationship between biomass and phenology also occurs at the molecular level, despite the lack of a common QTL for biomass and tipping time in our study (Additional file 2). The SNP on chromosome 3H 99 cM, which associated with digital biomass between 42 and 58 DAS, co-localized with the flowering time gene *HvCMF1* [50]*.* Moreover, the marker on chromosome 3H at 105.9 cM with significant biomass association at DAS 12 (Additional file 2) was reported to be related to heading time [46]. A major QTL for seedling biomass was detected on the short arm of 7H, corroborating the finding in the full barley panel (A.H. Abdel-Ghani, personal communication). In this same region, a QTL for the time of tipping and awn primordium stage was identified [40]. Thus, genetic factors driving phenology in barley contribute to the phenotypic variation in biomass at different time points.

Phenology affects biomass at individual developmental stages and contributes to the phenotypic variation of biomass development dynamics. This is reflected at the phenotypic level by a positive correlation between tipping time and inflection point amounting to *r* = 0.45. Moreover, the effects of phenology on biomass development are also visible at the molecular level; one of the QTL detected for inflection point co-localized with a well-known flowering gene. The SNP on chromosome 7H at 34 cM is part of a sequence contig from the cultivar *Morex* carrying *HvFT1/Vrn3* (M. Mascher, personal communication), an orthologue of *FT* [51]. *HvFT1* promotes flowering under long day conditions [35].

Early biomass was correlated with TKW. Despite that, none of the early biomass QTL co-located with previously reported QTL for TKW in the full panel of the investigated barley collection. However, one early biomass locus (3H, 105.9 cM) was in the same region as a QTL for TKW in a barley NAM-population [46].

Biomass generally increases with the number of tillers (Additional file 1: Figure S5), but not all tillers will develop inflorescences and become productive [52]. Therefore, selection for biomass traits that include loci associated with tillering should be handled with care. The phenotypic association between biomass and tiller number is reflected at the molecular level: Three biomass-associated SNPs were reported to be associated with tiller number in our or previous studies (Table 1).

Plant height was not assessed in the current study. Nevertheless, two of the seven biomass QTL and one QTL for inflection point co-localized with a known QTL for plant height (Table 1) identified in a different population [46]. Taken together, our results lend further strength to the hypothesis that genes related to phenology and morphology show pleiotropic effects on biomass development.

### Candidate genes potentially involved in biomass development

The SNP on chromosome 7H at 140.9 cM, which explained the largest proportion of genetic variance across the different time points, was also previously reported to be associated with shoot dry weight in seedlings [43]. A likely candidate gene, namely *HvDIM/HvDWF1*, is located in close proximity at 140.6 cM [53, 54]. In arabidopsis, the related *DIMINUTO/DWARF1* gene encodes a protein involved in steroid synthesis. The corresponding mutant, *dim,* is deficient in campesterol and brassinosteroids [55] which is caused by the inhibition of an early step in brassinosteroid biosynthesis that converts 24-methylenecholesterol to campesterol [56]. Brassinosteroids are growth-related hormones that regulate cell division, cell elongation, and photosynthesis, among other functions [57]. Brassinosteroids affect plant architecture traits such as height, leaf angle, tiller number, and grain size, thereby influencing yield [58]. Houston et al. [59] also reported additional candidate genes for biomass in this same region: one member of the Glycosyl Transferase family (*HvGsl5*), one member of the Glycosyl Hydrolase family (*GlbII*) and the gene *Sucrose Synthase II* (*HvSuSyII*), involved in the synthesis of cellulose [60, 61]. Another potential candidate at 140.7 cM is listed as an *ent-*copalyl diphosphate synthase [54], a precursor for gibberellins known to be involved in shoot growth [62, 63].

### Potential of marker-assisted selection for improved vegetative biomass

**Fig. 4:**
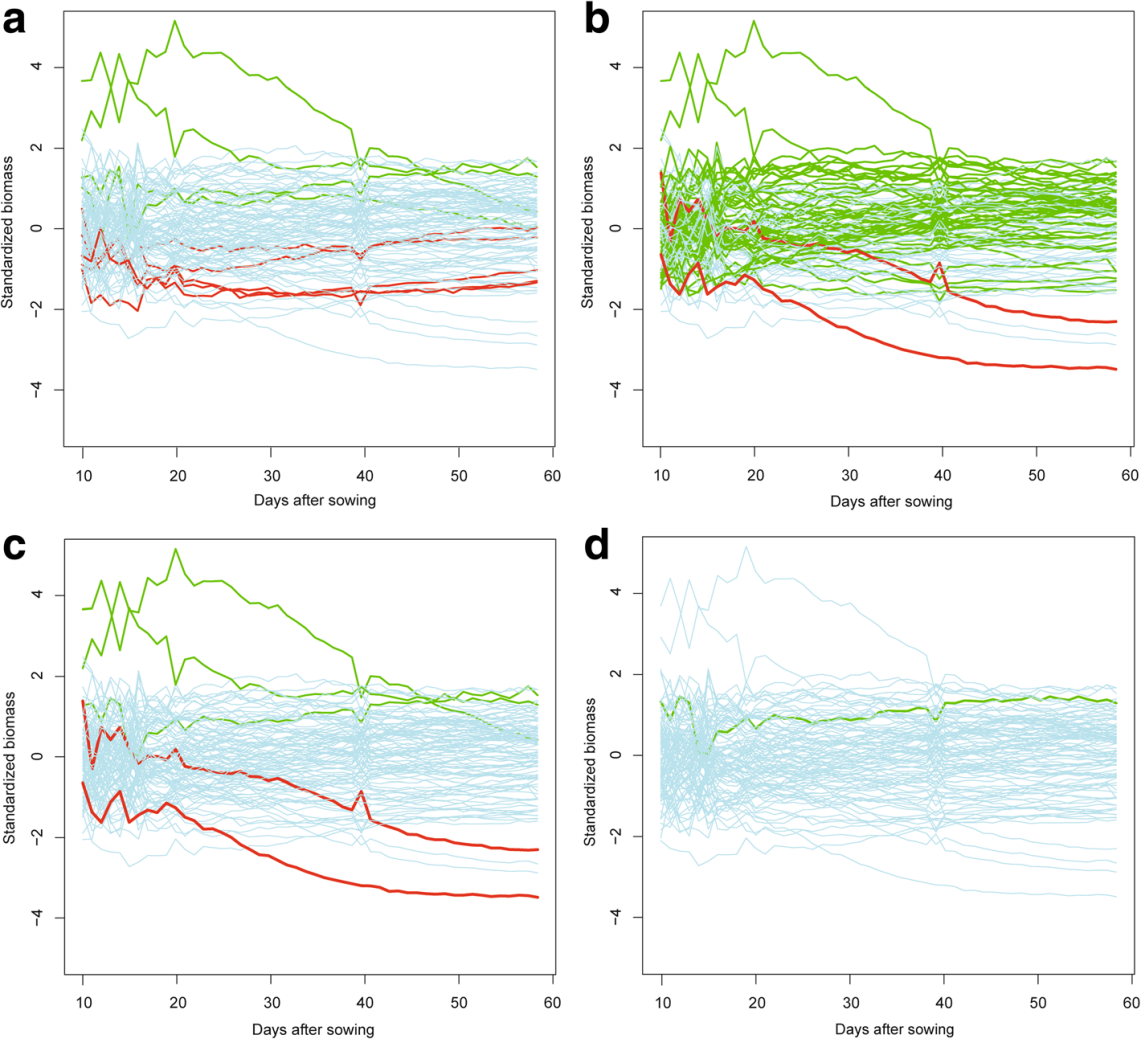
Standardized digital biomass of all barley accessions highlighting favorable and non-favorable QTL allele combinations over time. Biomass was standardized each day according to the population average. Values below zero indicate genotypes with biomass values lower than the average for the population; values above zero represent genotypes with biomass values higher than the average for the population. Genotypes that carry the positive marker alleles for each QTL set are highlighted in *green*, those carrying the non-favorable alleles are shown in *red*. The remaining allelic combinations for all other genotypes are shown in *grey* (**a**) QTL for early biomass (3 QTL) (**b**) QTL for late biomass (4 QTL) and (**c**) QTL for early and late biomass (7 QTL) (**d**) QTL for early and late biomass and for the inflection point (9 QTL)

Early vigor is hypothesized to be pivotal for seedling establishment and the promotion of increased final vegetative biomass [64]. To examine this hypothesis in more detail, we grouped the accessions according to their allelic state for each of the three early, and four medium-to-late biomass QTL. Identification of genotypes with increased seedling biomass was possible by selecting for favorable early QTL alleles (Fig. 4a), but identification of genotypes with a high biomass at reproductive stages was not successful using only QTL for late biomass (Fig. 4b). Interestingly, genotypes with very high late biomass were identified by selecting for favorable early and late biomass QTL (Fig. 4c). Moreover, we evaluated the potential of marker-assisted selection using information from all seven biomass QTL, in combination with the two QTL for inflection point (Fig. 4d). Only one genotype, which ranked amongst the lines with highest final biomass, combined the favorable alleles from all nine QTL (Fig. 4d) but no genotype combined all the unfavorable alleles. Hence, marker-assisted selection to identify increased biomass at the reproductive stage would benefit from the inclusion of QTL for biomass at the booting and seedling stage.

## Conclusions

This study demonstrates the potential of daily trait assessment to uncover the dynamics of trait relationships and to identify QTL for mapping. Our results show that biomass development during early and late growth stages is orchestrated by different QTL. Marker-assisted selection for late vegetative biomass is most effective by including favorable alleles from biomass QTL in both early and late vegetative stages. Using dynamic QTL for selection may enhance genetic gain for complex traits such as biomass or, in the future, grain yield. Our results also evaluated the genetic architecture of biomass development, and point at the impact of flowering time and plant morphology. To further refine biomass establishment QTL, future studies will benefit from the development and analysis of customized populations with reduced variation in flowering time, plant height and tillering. This study identified seven biomass QTL with large effects, three for early, one for medium, and three for late vegetative biomass accumulation. Looking ahead, fine mapping in bi-parental populations will reveal the genetic architecture and molecular basis of biomass formation under standardized conditions while field trial validation will assess the agronomic relevance of the present findings.

## Additional files

Additional file 1: Supplementary Data on barley collection, missing data points, phenotypic correlations, seasonal effects and phenology, map density and LD. The file contains supplementary **Tables S1-S4** and supplementary **Figures S1-S15**. (DOCX 9037 kb)

Additional file 2: Excel table with all SNPs that surpassed the FDR for at least one trait with mapping positions and –log(p)-values over all traits. (XLSX 46 kb)

## Abbreviations

BLUEs: Best linear unbiased estimates
DAS: Days after sowing
FDR: False discovery rate
GWAS: Genome-wide association studies
GxE: Genotype by environment interaction
LD: Linkage disequilibrium
QTL: Quantitative trait locus
SNP: Single-nucleotide polymorphism
TKW: Thousand-kernel weight
