# Supplementary material for: Genetic architecture and temporal patterns of biomass accumulation in spring barley revealed by image analysis
Source: BMC Plant Biol. 2017 Aug 10;17:137. doi: 10.1186/s12870-017-1085-4 (PMC5554006; doi:10.1186/s12870-017-1085-4)
Supplement: Supplementary file 1 — Supplementary Data on barley collection, missing data points, phenotypic correlations, seasonal effects and phenology, map density and LD. The file contains supplementary Tables S1-S4 and supplementary Figures S1-S15. (DOCX 9037 kb) [file 12870_2017_1085_MOESM1_ESM.docx]

**Genetic architecture and temporal patterns of biomass accumulation in spring barley revealed by image analysis**

*Kerstin Neumann, Yusheng Zhao, Jianting Chu, Jens Keilwagen, Jochen C. Reif, Benjamin Kilian, Andreas Graner*

Supplementary Data

[Barley collection 2](#_Toc486322567)

[Missing data points during image acquisition 2](#_Toc486322568)

[Phenotypic correlations of biomass to other traits 2](#_Toc486322569)

[Investigation of seasonal effects and influence of phenology on biomass 3](#_Toc486322570)

[Genetic map density and linkage disequilibrium 4](#_Toc486322571)

[Table S1 5](#_Toc486322572)

[Table S2 7](#_Toc486322573)

[Table S3 7](#_Toc486322574)

[Table S4 8](#_Toc486322575)

[Fig. S1 9](#_Toc486322576)

[Fig. S2 9](#_Toc486322577)

[Fig. S3 10](#_Toc486322578)

[Fig. S4 11](#_Toc486322579)

[Fig. S5 11](#_Toc486322580)

[Fig. S6 12](#_Toc486322581)

[Fig. S7 13](#_Toc486322582)

[Fig. S8 14](#_Toc486322583)

[Fig. S9 15](#_Toc486322584)

[Fig. S10 16](#_Toc486322585)

[Fig. S11 17](#_Toc486322586)

[Fig. S12 18](#_Toc486322587)

[Fig. S13 18](#_Toc486322588)

[Fig. S14 19](#_Toc486322589)

[Fig. S15 19](#_Toc486322590)

# **Barley collection**

The majority of barley genotypes (88) are coming from the Barley Core Collection (prefix BCC in Table S1) kept by the Leibniz-Institute of Plant Genetics and Crop Plant Research (IPK), while other nine accessions come directly from the genebank collection of IPK (prefix HOR in Table S1). Seeds from this collection are available upon request according to the regulations of the International Treaty for Plant Genetic Resources for Food and Agriculture (FAO) in combination with a standard Material Transfer Agreement (sMTA). Further, we added three reference barley cultivars: Barke, Arta and Tadmor. Barke is a listed cultivar from “Saatzucht Breun” (Amselweg 1, 91074 Herzogenaurach, Germany) and can be obtained from there. Seeds from Arta and Tadmor were received from the International Center for Agricultural Research in the Dry Areas (ICARDA).

# **Missing data points during image acquisition**

Due to technical problems or new installations and updates during the three experiments, incomplete data were obtained. In experiment 1 no images were acquired at DAS 11 and 14 and only incomplete series of images was retrieved on DAS 15 and 39. In experiment 2 no images were obtained at DAS 15 and in experiment 3 at DAS 13 and 19.

# **Phenotypic correlations of biomass to other traits**

Correlation of fresh weight (FW) measured at DAS 59 to digital biomass (DB) throughout the time course was increasing over time plateauing at R > 0.9 toward this final phase of the experiment (Fig S5a), reflecting again that later times points of DB are highly related to each other.

As especially early biomass may depend on the weight of the planted seed, we tested the correlation of DB over time to initial seed weight given as thousand kernel weight (Fig. S5a). We detected significant (P < 0.05) positive minor correlations from DAS 10 (R= 0.41) until DAS 25 (R = 0.20) with decreasing magnitude over time. No more significant correlations were observed after DAS 26.

The inflection point showed initially minor negative correlations to DB (Fig S5a), in particular during the period DAS 16 to 25 (R < -0.4). Afterwards it increased continuously until a significant albeit weak positive correlation was observed from DAS 55 on (at DAS 58 R = 0.27).

Medium to high correlations were observed between tiller number (T)N and DB (Fig. S5b). Significance started for early TN (DAS 27) at DAS 13, for medium TN (DAS 45) at DAS 26 and for late TN (DAS 58) at DAS 20. Highest correlations to DB were observed for early TN (maximum at DAS 33, R=0.75). In the middle of the experiment TN showed its highest correlation at DAS 37 and 38 (R=0.66). The weakest correlations were observed for late TN (maximum at DAS 39, R= 0.54).

Correlations between TN, IP, FW and TKW are summarized in Table S4. As for DB also FW was higher correlated with early and medium TN as with late TN. In accordance with late DB, IP showed a weak positive correlation to FW and further, a weak negative correlation to TKW.

# **Investigation of seasonal effects and influence of phenology on biomass**

Minor trends in biomass development were observed reflecting the different seasons during which the three experiments were performed (early summer, late summer, fall, Fig. S1). Especially in experiment 2 (summer) plants had a stronger biomass development. Differences between experiments depend further on the time course. Until DAS 47, significant differences between all three experiments were obtained by ANOVA and a Posthoc test, with experiment 3 having the lowest and experiment 2 the highest DB. But from DAS 47 onwards, experiments 1 and 3 formed one group while DB in experiment 2 remained significantly higher until the end. Seasonal differences were also observed for other traits. TN at DAS 27 and 45 was highest in experiment 2, in accordance with the overall higher biomass development (Fig. S2). Nevertheless, TN at DAS 58 of experiments 2 and 3 were similar, while TN in experiment 1 was significantly lower. By contrast, inflection point (IP) was earliest in the summer experiment 2 (45.1 DAS), and latest in autumn experiment 3 (49.9 DAS).

We observed a similar correlation pattern of biomass over time to tipping (Fig. S6) as detected for inflection point and biomass (Fig. S5a). But correlation coefficients of tipping and late DB were higher as for IP and DB.

# **Genetic map density and linkage disequilibrium**

The map saturation was sufficient with an average marker distance of 4.16 SNPs per cM and higher SNP density at centromeric regions. In total, nine gaps >5 cM were observed and only one gap > 10 cM located on 2HL spanning 11.8 cM (Fig. S7). A substantial amount of markers was mapping to identical genetic positions. Considering a window size of a 1cM, marker density per chromosome ranged between 1 and 160 SNPs per cM. The highest number of SNPs per window and chromosome was observed in the centromeric regions on 2H and 5H with a minimum of 24 SNPs and a maximum of 160 SNPs, respectively.

The average LD decay across all chromosomes in the panel of 99 spring barley accession was observed at 8 cM (Fig. S8) with the calculated threshold for r^2^ being 0.08. The LD decay varied between chromosomes (Fig. S9). Complete LD, i.e. r^2^ = 1, was detected only for 19 marker pairs beyond a genetic map distance of 1 cM, over the longest distance of 10.8 cM.

Table S1 Overview of the two-rowed barley germplasm with the respective Genebank accession number, region and country of origin and the biological status. The first three cultivars without accession number represent additional cultivars that are not part of the Genebank population. AM = American continent, EU = Europe, WANA = West Asia North Africa.

| **Accession name** | **Accession**  **number** | **Region of origin** | **Country of origin** | **Biological status** |
| --- | --- | --- | --- | --- |
| Arta | - | WANA | SYR | advanced/improved cultivar |
| Tadmor | - | WANA | SYR | advanced/improved cultivar |
| Barke | - | EU | DEU | advanced/improved cultivar |
| Apex | BCC1367* | EU | DEU | advanced/improved cultivar |
| Aramier | BCC1368 | EU | NLD | advanced/improved cultivar |
| Beatrice | BCC1370 | EU | FRA | advanced/improved cultivar |
| Beka | BCC1371 | EU | FRA | advanced/improved cultivar |
| Bielik | BCC1372 | EU | POL | advanced/improved cultivar |
| Blenheim | BCC1373 | EU | GBR | advanced/improved cultivar |
| Gambrinus | BCC1374 | EU | NLD | advanced/improved cultivar |
| Carlsberg II | BCC1376 | EU | DNK | advanced/improved cultivar |
| Ceres | BCC1377 | EU | FRA | advanced/improved cultivar |
| Claret | BCC1378 | EU | GBR | advanced/improved cultivar |
| Diamant | BCC1379 | EU | CZE | advanced/improved cultivar |
| Gavotte | BCC1380 | EU | FRA | advanced/improved cultivar |
| Georgie | BCC1381 | EU | GBR | advanced/improved cultivar |
| Golden Promise | BCC1382 | EU | GBR | advanced/improved cultivar |
| Golf | BCC1383 | EU | GBR | advanced/improved cultivar |
| Gryf | BCC1385 | EU | POL | advanced/improved cultivar |
| Haisa II | BCC1386 | EU | DEU | advanced/improved cultivar |
| Hebe | BCC1387 | EU | NLD | advanced/improved cultivar |
| Hunter | BCC1389 | EU | IRL | advanced/improved cultivar |
| Ingrid | BCC1390 | EU | SWE | advanced/improved cultivar |
| Isaria | BCC1391 | EU | DEU | advanced/improved cultivar |
| Kenia | BCC1392 | EU | DNK | advanced/improved cultivar |
| Mansholt | BCC1394 | EU | NLD | advanced/improved cultivar |
| Menuet | BCC1395 | EU | NLD | advanced/improved cultivar |
| Mette | BCC1396 | EU | SWE | advanced/improved cultivar |
| MFB 104 | BCC1397 | EU | HUN | traditional cultivar/landrace |
| MK 42 | BCC1398 | EU | HUN | breeding/research material |
| Nancy | BCC1399 | EU | SWE | advanced/improved cultivar |
| Natasha | BCC1400 | EU | FRA | advanced/improved cultivar |
| Ortolan | BCC1401 | EU | DEU | advanced/improved cultivar |
| Pallas | BCC1402 | EU | SWE | advanced/improved cultivar |
| Perun | BCC1403 | EU | DEU | advanced/improved cultivar |
| Plumage Archer | BCC1405 | EU | GBR | advanced/improved cultivar |
| Probstdorfer Adorra | BCC1407 | EU | AUT | advanced/improved cultivar |
| Proctor | BCC1408 | EU | GBR | advanced/improved cultivar |
| Quantum | BCC1409 | EU | AUT | advanced/improved cultivar |
| Rika | BCC1410 | EU | SWE | advanced/improved cultivar |
| Sissy | BCC1413 | EU | DEU | advanced/improved cultivar |
| Spartan | BCC1414 | EU | CZE | advanced/improved cultivar |
| Trumpf (Triumph) | BCC1417 | EU | DEU | advanced/improved cultivar |
| Tyra | BCC1418 | EU | DNK | advanced/improved cultivar |
| Union | BCC1419 | EU | DEU | advanced/improved cultivar |
| Valticky | BCC1421 | EU | CZE | advanced/improved cultivar |
| Varunda | BCC1422 | EU | NLD | advanced/improved cultivar |
| Volga | BCC1423 | EU | FRA | advanced/improved cultivar |
| Volla | BCC1424 | EU | DEU | advanced/improved cultivar |
| Wisa | BCC1425 | EU | DEU | advanced/improved cultivar |
| Chevallier | BCC1430 | EU | FRA | advanced/improved cultivar |
| Goldfoil | BCC1431 | EU | AUT | advanced/improved cultivar |
| Hana | BCC1432 | EU | CZE | advanced/improved cultivar |
| Heils Franken | BCC1433 | EU | DEU | advanced/improved cultivar |
| Kwassitzer Hanna | BCC1439 | EU | CZE | advanced/improved cultivar |
| Pammers Hohenauer Vollkorn | BCC1440 | EU | AUT | advanced/improved cultivar |
| Pflugs Intensiv | BCC1441 | EU | DEU | advanced/improved cultivar |
| Plumage | BCC1442 | EU | GBR | advanced/improved cultivar |
| Probsteiner Landgerste | BCC1443 | EU | DEU | advanced/improved cultivar |
| Slovensky 802 | BCC1444 | EU | CZE | advanced/improved cultivar |
| Souche 142 Strotzheim | BCC1445 | EU | FRA | breeding/research material |
| Risk | BCC1457 | EU | RUS | advanced/improved cultivar |
| Ilmen | BCC1458 | EU | RUS | advanced/improved cultivar |
| Omskij 80 | BCC1461 | EU | RUS | advanced/improved cultivar |
| Primorskij 89 | BCC1463 | EU | RUS | advanced/improved cultivar |
| Doneckij 650 | BCC1465 | EU | UKR | advanced/improved cultivar |
| Odesskij 100 | BCC1466 | EU | UKR | advanced/improved cultivar |
| Zhodinskij 5 | BCC1467 | EU | BLR | advanced/improved cultivar |
| Tselinij 213 | BCC1468 | WANA | KAZ | advanced/improved cultivar |
| Granal | BCC1469 | WANA | KAZ | advanced/improved cultivar |
| Auksinjai | BCC1472 | EU | LTU | advanced/improved cultivar |
| K 16411 | BCC1480 | EU | RUS | traditional cultivar/landrace |
| K 3222 | BCC1481 | EU | RUS | traditional cultivar/landrace |
| Vjatich | BCC1482 | EU | RUS | advanced/improved cultivar |
| K 4511 | BCC1483 | EU | RUS | traditional cultivar/landrace |
| K 21820 | BCC1487 | EU | RUS | traditional cultivar/landrace |
| K 11749 | BCC1497 | WANA | KGZ | traditional cultivar/landrace |
| Odesskij 36 | BCC1506 | EU | UKR | advanced/improved cultivar |
| Bavaria (Ackermanns Bavaria) | BCC1524 | EU | DEU | advanced/improved cultivar |
| HOR 753 | BCC1566 | EU | GRC | traditional cultivar/landrace |
| HOR 10555 | BCC1589 | EU | ITA | traditional cultivar/landrace |
| IG 31444 | BCC192 | WANA | SYR | traditional cultivar/landrace |
| IG 31513 | BCC195 | WANA | SYR | traditional cultivar/landrace |
| Fu 8 | BCC432 | EA | CHN | breeding/research material |
| Hyanmaeg | BCC675 | EA | KOR | advanced/improved cultivar |
| AC Obow | BCC801 | AM | CAN | advanced/improved cultivar |
| Compana | BCC847 | AM | USA | advanced/improved cultivar |
| Gobernadora | BCC869 | AM | ME | advanced/improved cultivar |
| Libra | BCC899 | AM | CHL | advanced/improved cultivar |
| Manley | BCC903 | AM | CAN | advanced/improved cultivar |
| Sanalta | BCC929 | AM | CAN | advanced/improved cultivar |
| MR 1/13 | HOR11370 | WANA | ISR | breeding/research material |
| MR 3/51 | HOR11371 | WANA | ISR | breeding/research material |
| W 23833/8108 | HOR11372 | WANA | ISR | breeding/research material |
| W 23833/2196 11 | HOR11373 | WANA | ISR | breeding/research material |
| W 23829/8039 11 | HOR11374 | WANA | ISR | breeding/research material |
| HOR 1391 | HOR1391 | EU | ROM | traditional cultivar/landrace |
| HOR 8050 | HOR8050 | WANA | TUR | traditional cultivar/landrace |
| HOR 8113 | HOR8113 | WANA | TUR | traditional cultivar/landrace |
| HOR 8160 | HOR8160 | WANA | TUR | traditional cultivar/landrace |

*BCC1367 had to be excluded from all genotypic analysis

Table S2 Time schedule of the phenotyping experiments

| **Experiment** | **Start of HTP experiment** | **End of HTP experiment** |
| --- | --- | --- |
| 1 | 08.05.2012 | 05.07.2012 |
| 2 | 20.07.2012 | 16.09.2012 |
| 3 | 25.09.2012 | 25.11.2012 |

Table S3 Descriptive statistics of the phenotypic BLUEs values over all experiments for the barley association panel for the first and last day of biomass evaluation (DB 10 and 58) in 10^6^ Voxel, TN at 27, 45 and 58 days after sowing, FW at 59 days after sowing in g, IP in days after sowing, initial TKW of seed source in g and the days until tipping (BBCH 49; TP) based on 93 accessions evaluated in the first two experiments.

| **Trait** | **Minimum** | **Maximum** | **Mean** | **SD** | **CV (%)** |
| --- | --- | --- | --- | --- | --- |
| DB 10 | 0.032 | 0.087 | 0.052 | 0.010 | 18.4 |
| DB 58 | 14.2 | 113.4 | 81.2 | 19.2 | 23.7 |
| TN 27 | 2.5 | 8.9 | 5.7 | 0.9 | 15.5 |
| TN 45 | 5.8 | 28.3 | 16.7 | 3.5 | 20.7 |
| TN 58 | 7.6 | 40.2 | 20.9 | 4.7 | 22.7 |
| FW 59 | 46.9 | 158.6 | 124.9 | 19.1 | 15.3 |
| IP | 43.6 | 52.1 | 47.5 | 1.7 | 3.5 |
| TKW | 42.2 | 63.3 | 50.0 | 4.3 | 8.6 |
| TP | 32.9 | 57.0 | 49.6 | 4.9 | 9.8. |

Table S4 Correlations of IP, TN at DAS 27, 45 and 58, FW and TKW based on the overall BLUEs. Pearson correlation coefficient R is presented if p<0.05. Non-significant correlation = n.s.

|  | **IP** | **TN 27** | **TN 45** | **TN 58** | **FW** | **TKW** |
| --- | --- | --- | --- | --- | --- | --- |
| **IP** | 1 |  |  |  |  |  |
| **TN 27** | -0.23 | 1 |  |  |  |  |
| **TN 45** | n.s. | 0.79 | 1 |  |  |  |
| **TN 58** | n.s. | 0.65 | 0.95 | 1 |  |  |
| **FW** | 0.22 | 0.58 | 0.59 | 0.48 | 1 |  |
| **TKW** | -0.32 | n.s. | n.s. | n.s. | n.s. | 1 |


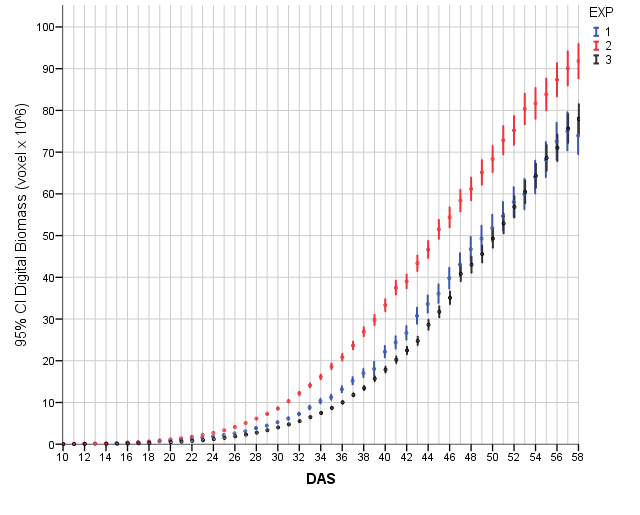


Fig. S1 Development of digital biomass over time in the course of three independent experiments from 10 to 58 days after sowing (DAS). The error bars represent the 95% confidence interval (CI) based on the BLUEs of single experiments of 100 barley genotypes.

Fig. S2 Boxplots of tiller number (TN) at DAS 27, 45 and 58 and inflection point (IP) for three experiments based on the BLUEs of 100 barley genotypes.


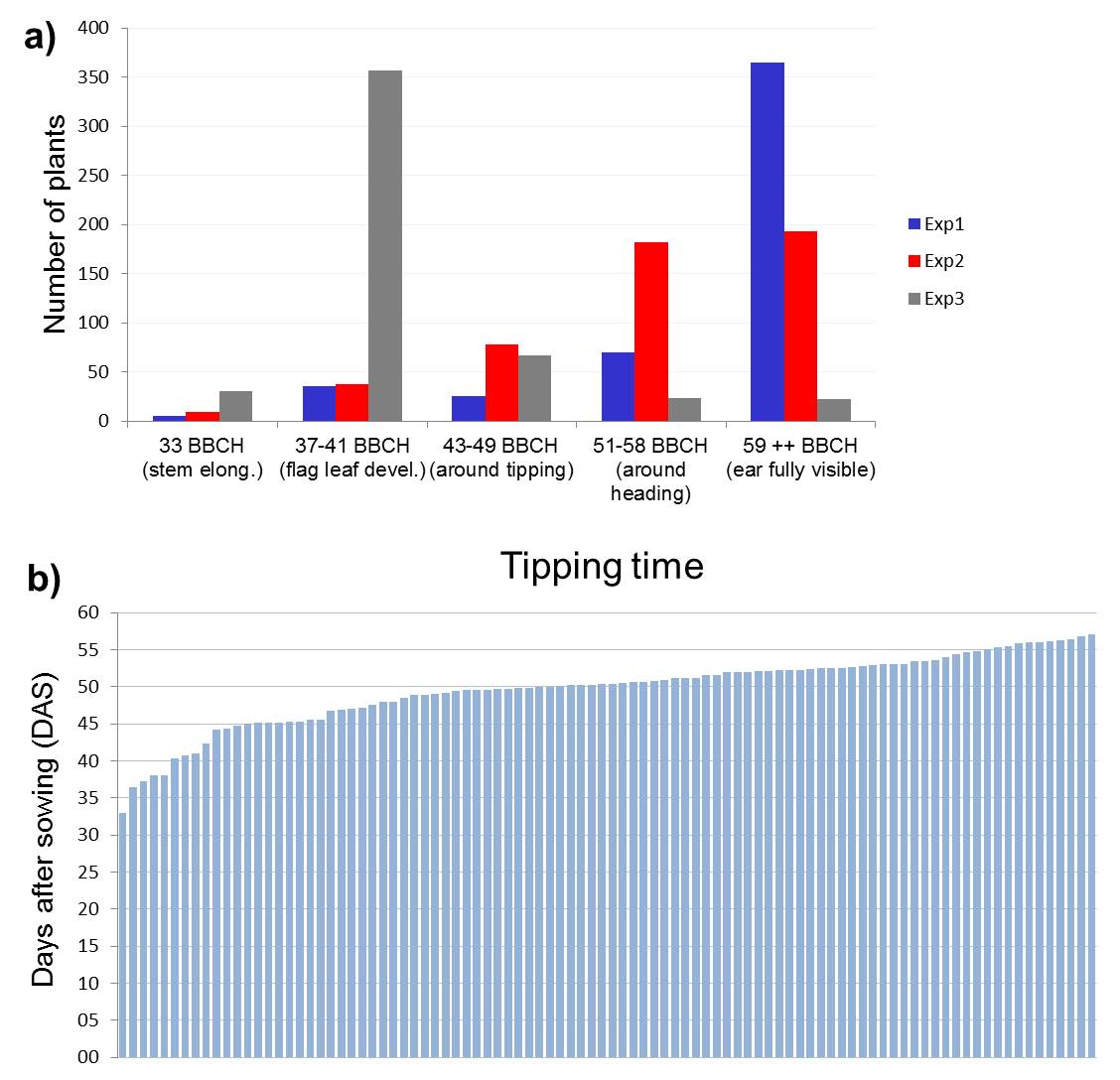


Fig. S3 Phenology evaluation **a)** Evaluation of growth stages according to the BBCH-scale (Lancashire *et al.* 1991; Witzenberger *et al.* 1989) determined from raw imagines at the last day of each experiment (DAS 58) **b)** Overview of exact tipping time (BBCH 49) in the barley panel. Genotypes are sorted ascending to their BLUES-values across experiments.


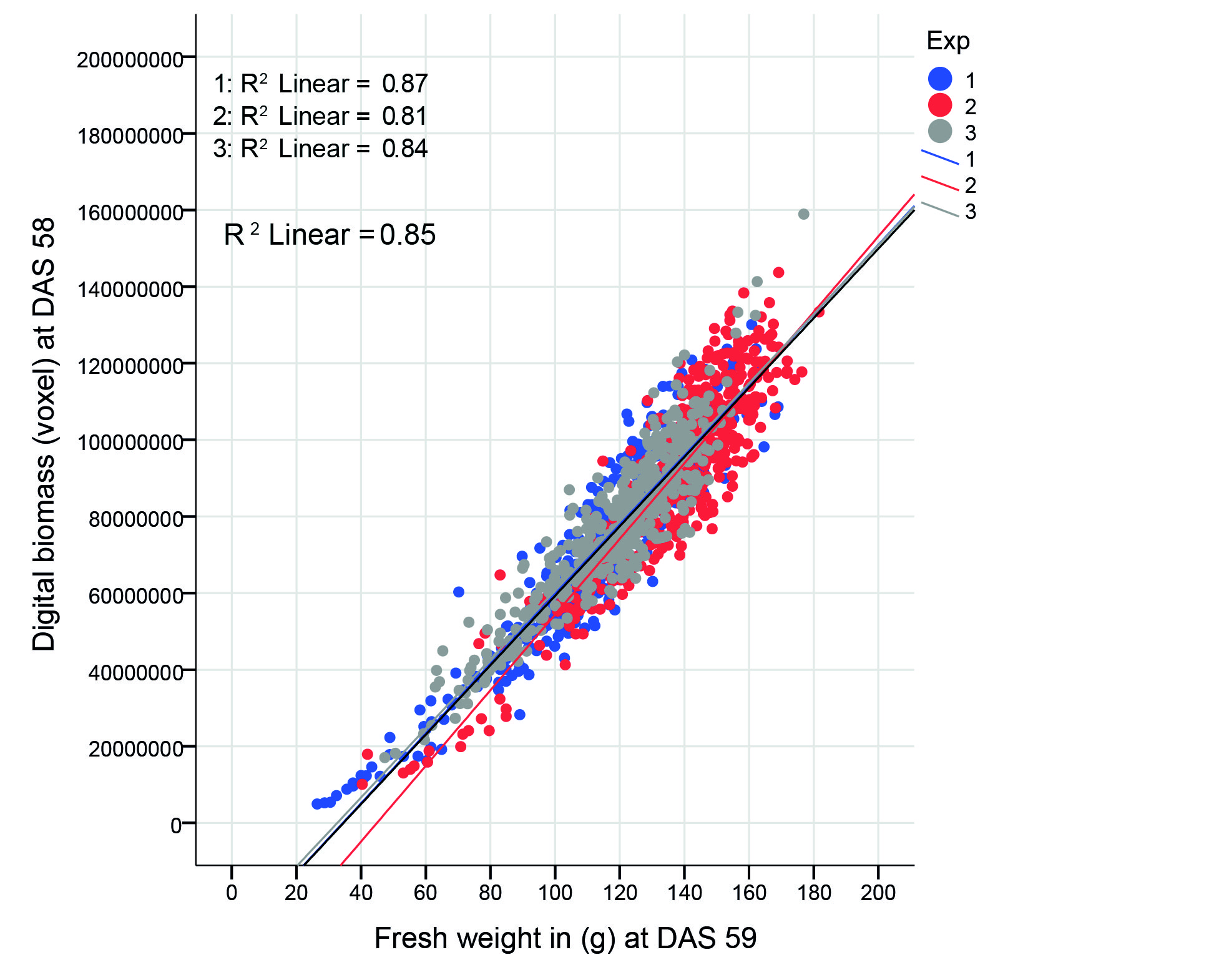


Fig. S4 Scatterplot of FW and DB for three experiments (1-blue, 2-red, 3-grey) with coefficients of determination (R^2^)within each experiment and the overall R^2^ value.

Fig. S5 Correlations of **a)** FW estimated at experiment end, TKW, IP to DB over time in days after sowing (DAS); and **b)** of TN at 3 time points (27, 45, 58) to DB over time. Colored vertical lines indicate the DAS where the correlation coefficient r is highest.


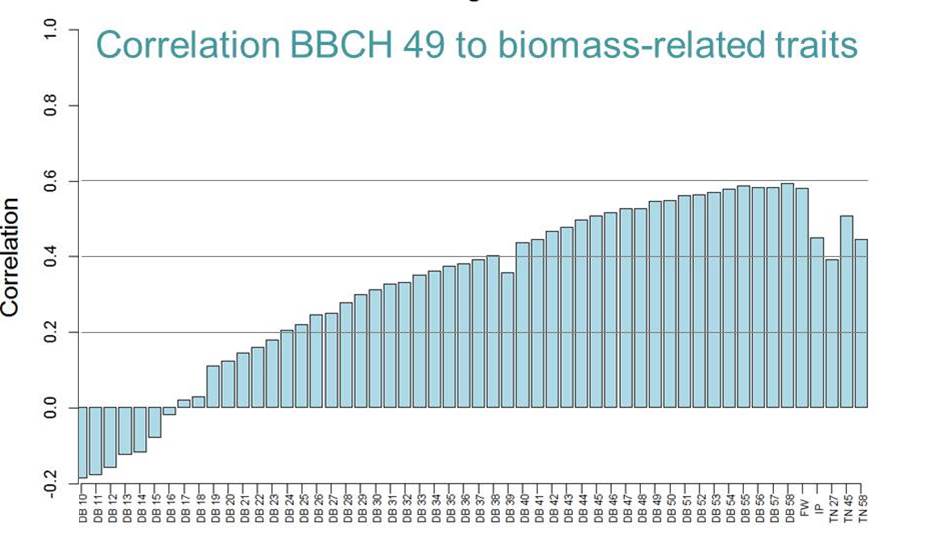


Fig. S6 Pearson correlation coefficient between tipping time (BBCH 49) and digital biomass (DB) over time, fresh weight (FW), inflection point (IP), tiller number (TN) over time.

Fig. S7 Map density across all seven barley chromosomes. Number of SNPs (y-axes) is plotted for the genetic position (x-axes) with a window size of 1 cM and a step size of 1 cM. For a genetic position of x cM, we count the SNPs in the interval from x-0.5 to x+0.5 cM. The number of SNPs is given for the cM window with the highest number of SNPs for each chromosome. The position of centromeres is indicated by red asterisks.


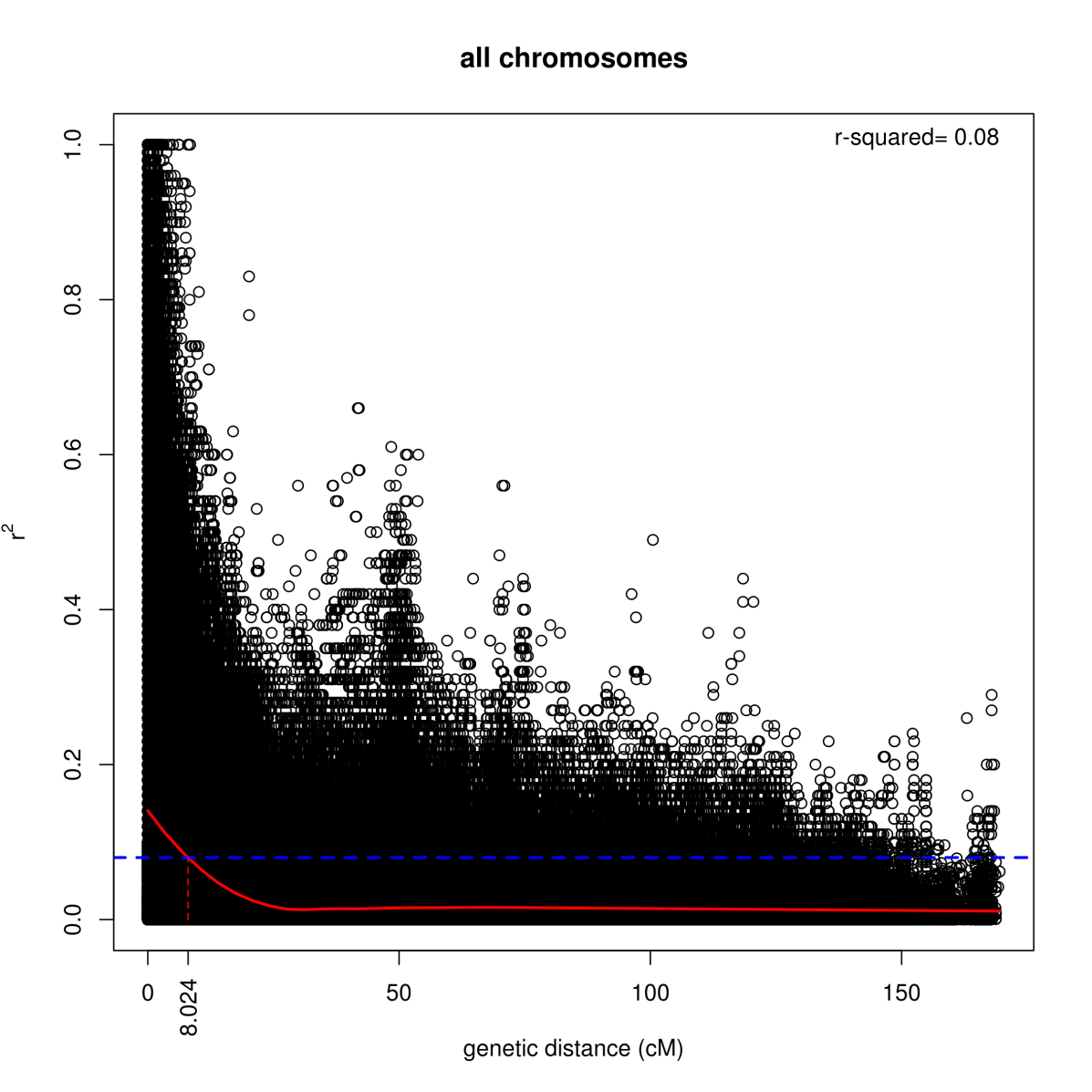


Fig. S8 Overall LD decay within the barley association panel. The calculated threshold of 95% percentile of unlinked marker pairs (distance > 50 cM) r^2^ = 0.08 is indicated by the dashed blue line, while the red solid line represents the fitted LOESS curve. The red vertical dashed line indicates the crossing of LOESS curve and the threshold line the cM distance of the cutting point is given below.

Fig. S9 Linkage disequilibrium decay for individual barley chromosomes. The calculated threshold of 95% percentile of unlinked marker pairs distance > 50 cM, the r^2^ value given for each chromosome in the upper right corner) is indicated by the dashed blue line, while the red line represents the fitted LOESS curve. The red vertical dashed line indicates the crossing of LOESS curve and the threshold line, the cM distance of the cutting point is given below.

Fig. S10 Scatterplot of first two principal components (PC) with the region of origin highlighted in different colors, in brackets is given the amount of how much variation is explained by each PC. The two American continents are indicated in green (AM), East Asia in yellow (EA), Europe in blue (EU) and the region of West Asia and North Africa in red.


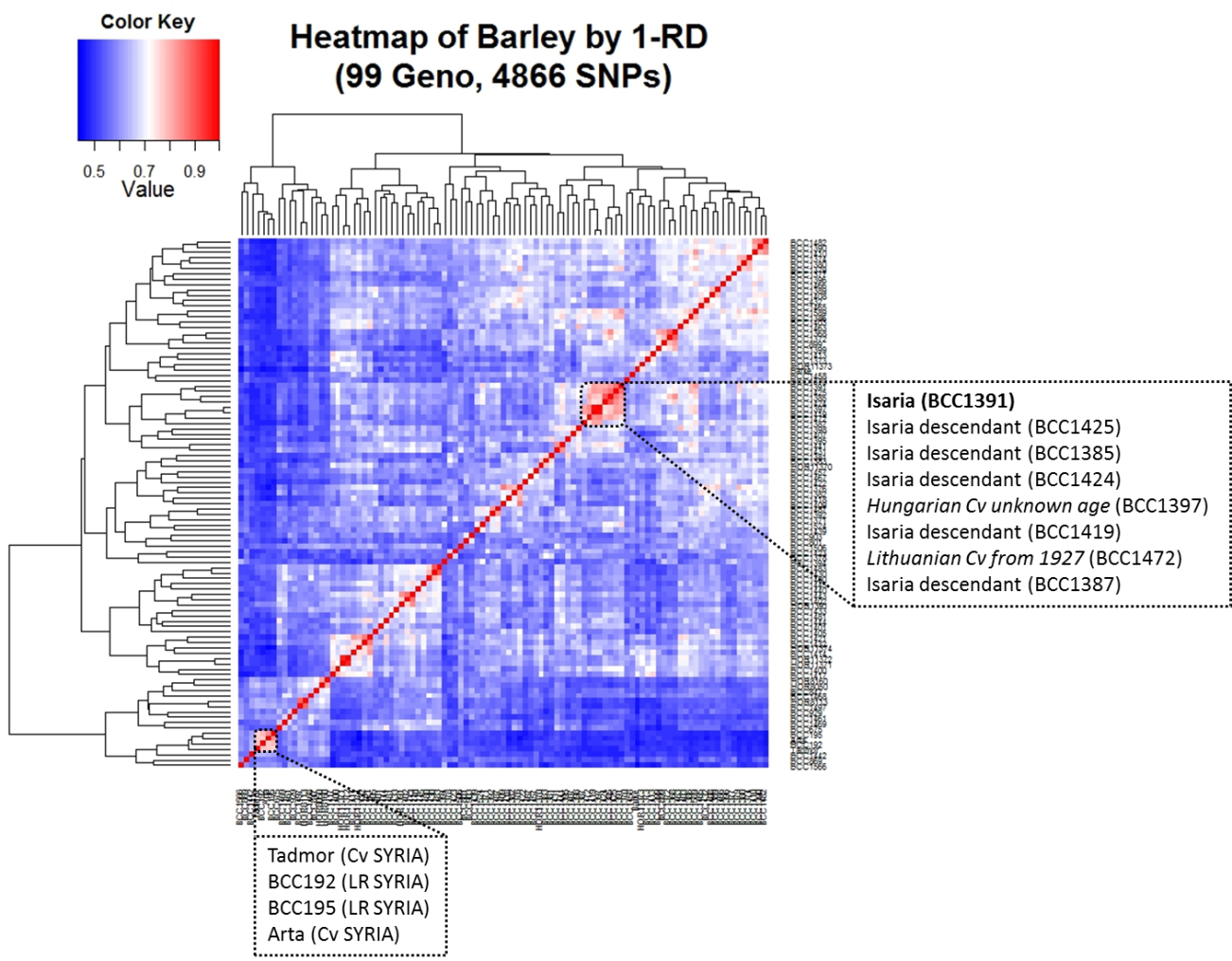


Fig. S11 Heatmap of relatedness estimated by Rogers’ distances (RD) between all barley genotypes. Values are adjusted by subtraction of 1 minus RD and are based on 4,866 polymorphic SNPs. Two examples of family structure are indicated by dashed lines: the bigger cluster is the “Isaria”- cluster (German cultivar from 1924), the smaller cluster refers to cultivars (Cv) and landraces (LR) from Syria.

Fig. S12 Manhattan plot of genome-wide association mapping results for DB of DAS 58 and FW at DAS 59. The red asterisks on chromosomes 3H, 6H and 7H denote two identical SNPs, respectively, for FW and DB58 passing the FDR (<0.1). Numbers from 1 to 7 at the top refer to the seven barley chromosomes.

Fig. S13 Manhattan plot of genome-wide association results for IP. The loci passing the false discovery rate (<0.1) are indicated by a red asterisk. Numbers from 1 to 7 at the top refer to the seven barley chromosomes.

**
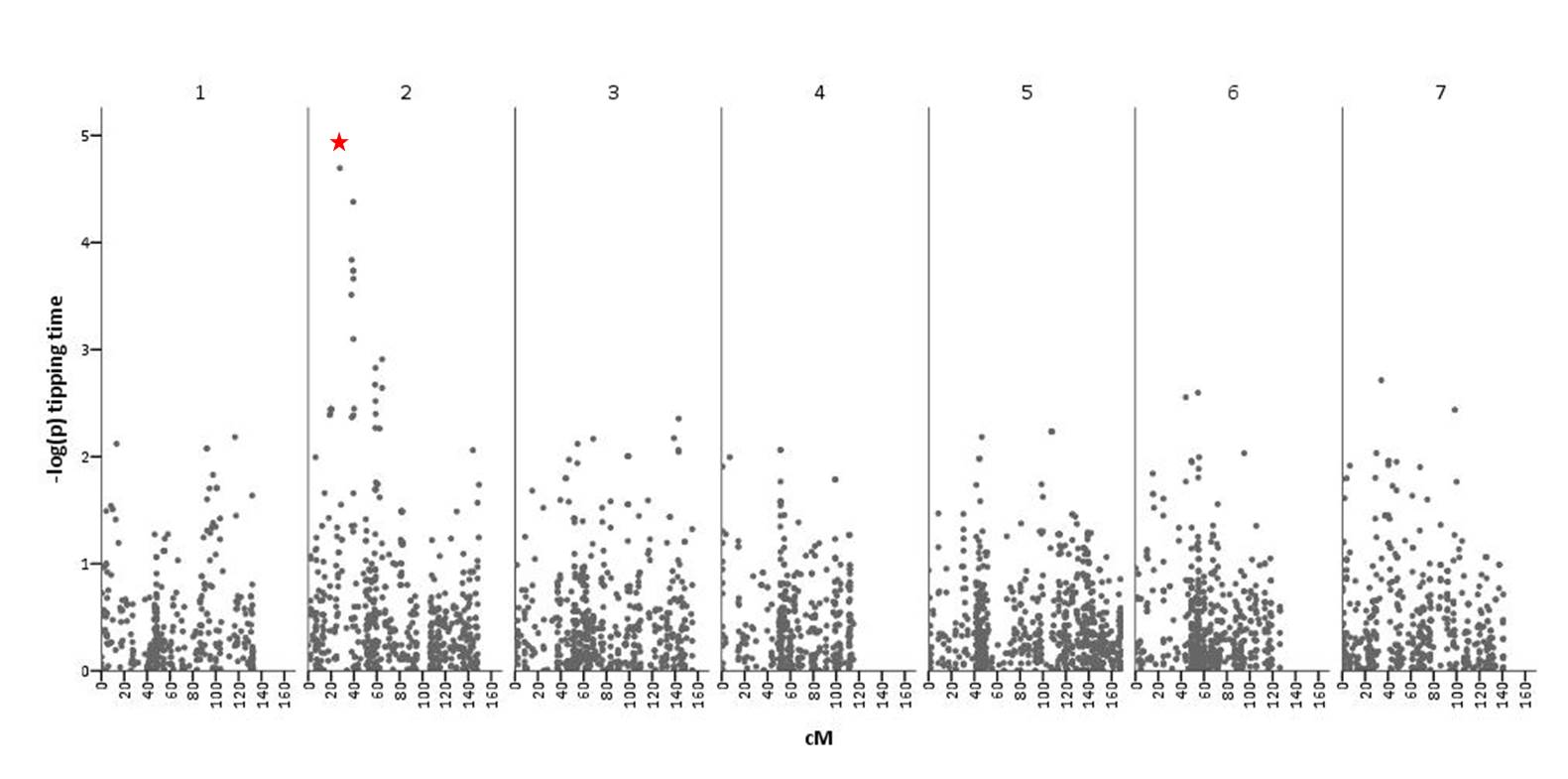
**

Fig. S14 Manhattan plot of genome-wide association mapping results for tipping time. The loci passing the false discovery rate (<0.1) are indicated by a red asterisk. Numbers from 1 to 7 at the top refer to the seven barley chromosomes.

Fig. S15 Manhattan plot of genome-wide association mapping results for tiller number (TN) at DAS 27, 45 and 58. The loci passing the false discovery rate (<0.1) are indicated by a red asterisk. Numbers from 1 to 7 at the top refer to the seven barley chromosomes.
